# Home monitoring of coronavirus disease 2019 patients in different phases of disease

**DOI:** 10.1097/MCP.0000000000000964

**Published:** 2023-05-09

**Authors:** Kalle Majoor, Adriane D.M. Vorselaars

**Affiliations:** a Department of Internal Medicine, Isala, Zwolle; b Division of Heart and Lungs, University Medical Center Utrecht; c Interstitial Lung Diseases Center of Excellence, Department of Pulmonology, St Antonius Hospital, Nieuwegein, The Netherlands

**Keywords:** coronavirus disease 2019, home monitoring, telemonitoring, virtual ward

## Abstract

Various home monitoring programs have emerged through the COVID-19 pandemic in different phases of COVID-19 disease.

**Recent findings:**

The prehospital monitoring of COVID-19-positive patients detects early deterioration. Hospital care at home provides early discharge with oxygen to empty hospital beds for other patients. Home monitoring during recovery can be used for rehabilitation and detection of potential relapses. General goals of home monitoring in COVID-19 are early detection of deterioration and prompt escalation of care such as emergency department presentation, medical advice, medication prescription and mental support. Due to the innovations of vaccination and treatment changes, such as dexamethasone and tocilizumab, the challenge for the healthcare system has shifted from large numbers of admitted COVID-19 patients to lower numbers of admitted patients with specific risk profiles (such as immunocompromised). This also changes the field of home monitoring in COVID-19. Efficacy and cost-effectiveness of home monitoring interventions depend on the costs of the intervention (use of devices, apps and medical staff) and the proposed patient group (depending on risk factors and disease severity).

**Summary:**

Patient satisfaction of COVID-19 home monitoring programs was mostly high. Home monitoring programs for COVID-19 should be ready to be re-escalated in case of a new global pandemic.

## INTRODUCTION

**FIGURE 1:**
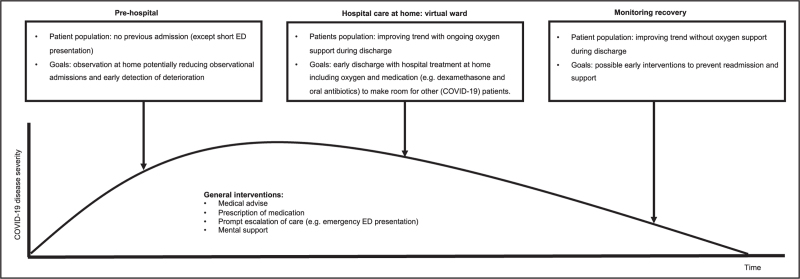
COVID-19 home monitoring in different phases of the disease. Individual COVID-19 disease severity determines the type of home monitoring. In the prehospital phase, meaning no previous admission except for an emergency department presentation, symptoms are mild to moderate, and patients are monitored for observation of deterioration. This possibly reduces observational admissions. Early discharge of improving COVID-19 patients with oxygen and medication including dexamethasone and antibiotics potentially shortens admission duration. Discharge of recovering patients without oxygen support with home monitoring gives patients support and detects early deterioration. ED, emergency department.

The severe acute respiratory syndrome coronavirus-2 (SARS-CoV-2) spread from December 2019 throughout the world and became a pandemic. SARS-CoV-2 causes coronavirus disease 2019 (COVID-19), which has substantial morbidity and mortality [1]. From the beginning of the pandemic, COVID-19 challenged the existing healthcare system in different ways. Face-to-face consultations had to be reduced to prevent virus transmissions without comprising healthcare services. Second, the burden of COVID-19-related care resulted in shortage of hospital beds and available personnel. Therefore, emergency departments had to perform strict triage for admission. In addition, hospitals started to perform early discharge whenever possible. The WHO urged the use of home monitoring (see Table 1 for alternative terms) to tackle these challenges [2]. Home monitoring facilitates communication between healthcare professionals and patients at a distance. Before the COVID-19 pandemic, home monitoring had proven effectiveness in different diseases, for example, in reducing mortality in heart failure patients [3]. Since the COVID-19 pandemic, home monitoring got a tremendous boost for many diseases including COVID-19 [4]. Home monitoring projects emerged for three different phases of the COVID-19 disease. First, before hospital admission, COVID-19 patients can be monitored at home to prevent observational admission and detect early deterioration. Second, home monitoring facilitates early discharge of recovering patients with oxygen and medication. Finally, recovery can be monitored. This narrative review will discuss home monitoring in COVID-19 patients in these different phases since the beginning of the COVID-19 pandemic (see Fig. 1). 

**Box 1:**
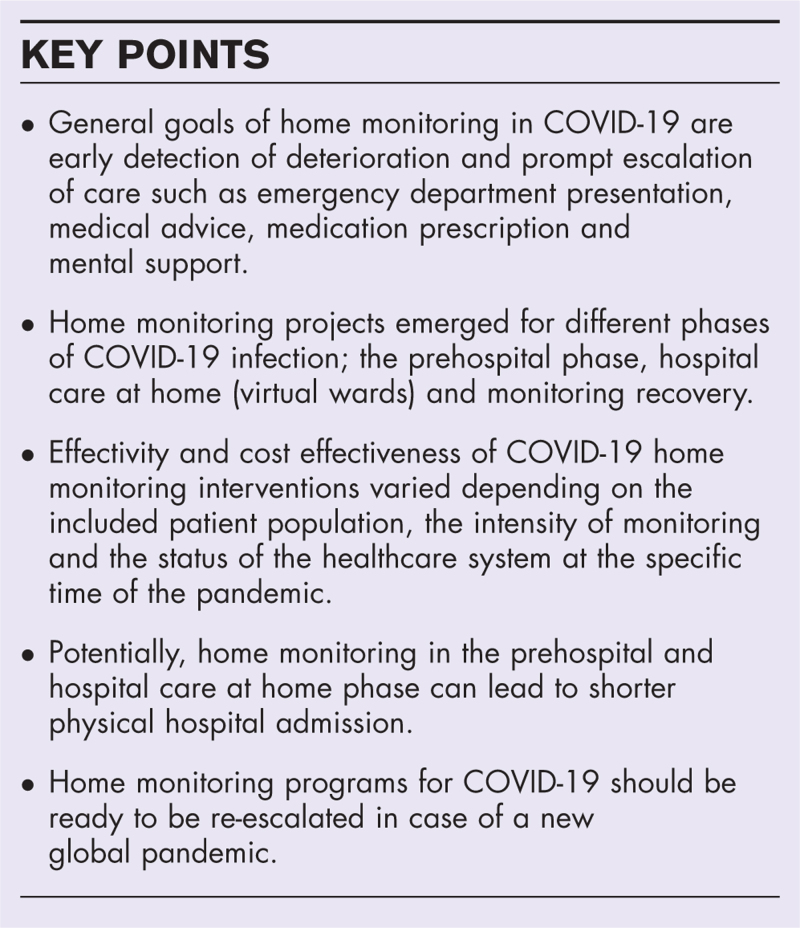
no caption available

**Table 1:** Different terms used for remote monitoring during the COVID-19 pandemic

Different terms used for remote monitoring during the COVID-19 pandemic
Home monitoring
Remote (patient) control
Telehealth
Telemedicine
Virtual ward
Mobile Health (m-Health)
Telemonitoring

## PREHOSPITAL

The majority of home monitoring for COVID-19 was performed in the prehospital phase, especially during the first wave in early 2020. This included asymptomatic or mild COVID-19 patients without previous hospital admission except a short stay at the emergency department. Complaints of COVID-19 are largely unspecific and similar to other respiratory viruses with fever, cough and shortness of breath [1]. COVID-19 has an unpredictable disease course. Therefore, frequent monitoring of signs and symptoms after a positive test was essential. Home monitoring facilitated this process and replaced admission for observation of symptoms. Early detection of deterioration could possibly prevent delay of treatment and favourably alter disease trajectory. Therefore, the United Kingdom started Oximetry@home early in the COVID-19 pandemic. Proven or suspected COVID-19 patients received a pulse oximeter to measure oxygen saturation and heart rate and communicated with healthcare professionals about their symptoms. Decline in oxygen saturation or clinical situation could lead to escalation of care. Hospital presentations and mortality in the monitoring program were low and, therefore, deemed well tolerated. Baseline characteristics showed mild disease activity with oxygen saturation of 95% or more in 85% of patients. Thirty-five percent of patients were under 65 years of age and had no comorbidities. These baseline characteristics could explain the low complications rates. This study lead to nationwide implementation of the Oximetry@home program (see Table 2) [5]. Prehospital home monitoring was effective in a recent large retrospective study in the United States. After a positive SARS-CoV-2 test, 9278 adult patients (mean age 46 years) received an invitation to participate in home monitoring, resulting in 5364 activations. Activation of home monitoring resulted in lower hospitalization (2.4 vs 3.9%), ICU admissions (0.3 vs. 1.1%) and 90-day mortality (0.2 vs. 0.6%) compared with patients who did not activate the system. After propensity score matching, the odds ratio for hospitalisation remained lower (0.68; 95% CI 0.54–0.86; *P* = 0.001) [6] (see Table 2). The Mayo Clinic home monitoring system for high-risk COVID-19 patients showed comparable results. After propensity score matching, engaged patients had significant lower hospitalization (13.7 vs. 18%), shorter admission (6.7 vs. 8.2%) and lower mortality (0.5 vs. 1.7%). Interestingly, home monitoring resulted in saving $1259 per patient during the 30-day follow-up period [7^▪▪^] (see Table 2). Australia started a nationwide funding for telehealth early in the COVID-19 pandemic. This resulted in many home monitoring systems [8]. As an example, a virtual hospital in Sydney monitored 162 low-risk COVID-19 patients (median age 38 and no severe comorbidities) directly after a positive test. Three patients were admitted and no deaths occurred. Therefore, the authors concluded this model was safe [9]. Prehospital monitoring can also be used in specific high-risk groups to screen for early deterioration. As an example, monitoring of 92 pregnant COVID-19-positive patients with mild symptoms was safe because of low complication rates and early detection of deterioration [10]. To conclude, home monitoring in the prehospital phase of COVID-19 seems safe and showed effectiveness on reducing duration of hospitalization, mortality during 30-day follow-up and cost effectiveness. However, the majority were retrospective studies and included healthy, young patients, and lacked a control group.

**Table 2:** Overview of important articles

	Population	Design	Main outcomes and notes
Assessing the safety of home oximetry for COVID-19: a multisite retrospective observational study, Clarke *et al.*, 2021, United Kingdom [5]	908 confirmed or suspected COVID-19 patients. 65% of patients were 65 years of age or younger and had no comorbidities.85% of patients had oxygen saturation at rest of 95% or more.	Retrospective, uncontrolled study.Patients were enrolled following referral by primary care, emergency department, or after hospital admission.They measured oxygen saturation on top of standard care and communicated with the regular healthcare professional. Data were transferred via app-based platforms and paper diaries.	52 (5.7%) presented to the hospital and 28 (3.1%) died following enrolment. Mortality after hospital admission was highest. The system was deemed safe because of low complication rates. However, this could be explained inclusion of young patients without comorbidities and good oxygen saturation at rest.
Hospitalization Outcomes Among Patients With COVID-19 Undergoing Remote Monitoring, Crotty *et al.*, 2022, United States [44^▪▪^].	9278 invited adult confirmed COVID-19 patients. 5364 activations (mean age 46 years, 65% women).Exclusion criteria were admission within 24 h after starting the home monitoring and patients with asymptomatic positive test, because this was often before a planned admission.	Retrospective observational cohort study. They performed daily temperature and oxygen saturation measurements and sent data via a web or mobile application.Patients had the option to send open text message. Data were checked by nurses 24 h a day, 7 days a week. Dyspnoea or fever created alarms. Education to patients was automated.They performed propensity score matching.	Differences were seen in the group that activated the program compared with patients who did not activate: admission rates 2.4 vs 3.9%, admission duration 4.4 vs 7.1%, ICU admission 0.3 vs 1.1%, and 90-day mortality 0.2 vs. 0.6%.After propensity score matching, the activation of RPM was associated with a lower odds ratio for hospitalization (0.68; 95% CI, 0.54–0.86; *P* = 0.001).
Impact of a high-risk, ambulatory COVID-19 remote patient monitoring program on utilization cost and mortality, Haddad *et al.*, 2022, United States [7^▪▪^].	5796 enrolled patients. Mean age 63 years, 51% women and 82% non-Hispanic white. The study included 1128 pairs of home monitoring engaged compared with nonengaged matched controls.	Retrospective cohort analysis. Oxygen saturation, blood pressure, temperature and symptoms were monitored two to four times a day. Data were registered with a tablet directly connected to the electronic health record and checked by nurses 24 h per day, 7 days a week.Follow-up was 30 days.Propensity score matching was performed.	Favourable results were seen in the monitoring compared with controls: hospitalisation: 13.7 vs. 18% (*P* = 0.01), prolonged hospitalization 3.5 vs. 6.7% (*P* = 0.001), ICU admission 2.3 vs. 4.2% (*P* = 0.01), 30-day mortality 0.5 vs 1.7%, odds ratio 0.31; (95% CI 0.12–0.78; *P* = 0.01), cost of care per patient $2306.33 vs. $3565.97 (*P* = 0.04).
Remote Hospital Care for Recovering COVID-19 Patients Using Telemedicine: A Randomised Controlled Trial, Van Goor *et al.*, 2021, Netherlands [14].	They included 62 confirmed adult COVID-19 patients (mean age 55 years) with improving trend. Exclusion criteria were patients suffering of dementia or other illness that limited compliance to home monitoring.	Nonblinded randomized controlled trial.Regular hospital care was compared with home monitoring with oxygen (maximum 3 l/min) and oral medication including dexamethasone and antibiotics.Randomization 1 : 1. 30-day follow-up. They measured oxygen saturation and temperature and filled out symptoms via a mobile application. Phone call was performed by medical students.	The hospital free days were 1.7 days (*P* = 0.112) higher with home monitoring. This was probably not significant because of group size. In addition, patients in the control group were also discharged with oxygen at home without home monitoring during the study resulting in a reduced difference. Oxygen therapy was longer in the home monitoring group (6.7 vs. 3.4 days, *P* = 0.101) and longer care under hospital responsibility (14 vs. 10 days, *P* = 0.028).
A Covid -19 Virtual Ward Model: A Preliminary Retrospective Clinical Evaluation From a UK District General Hospital, O’Malley *et al.*, 2022, United Kingdom [15^▪^].	43 confirmed COVID-19 patients (mean age 58 years) were discharged early with an improving trend.	Retrospective, uncontrolled study. The maximal oxygen support was 4 l/min. Patients received oral medication including dexamethasone and antibiotics. The general practitioner was responsible for the home monitoring.Temperature, heart rate, blood pressure and SpO_2_ measured thrice a day. A mobile device was used to fill out recordings. Phone calls, video calls or physical assessment were performed. Goal: every 24–48 h 1 l/min oxygen tapering if no dyspnea or SpO_2_ < 94%.	72% received supplemental oxygen.Four (9%) were readmitted because of hypoxia. All were identified with the home monitoring system. Mean hospital stay was 10.3 days with monitoring compared with 11.9 days for other COVID-19 patients in the same period. Mean duration of monitoring 13.7 and 11.6 days of oxygen at home.They included no data of baseline characteristics of the COVID-19 patients during same period, which may have led to confounding.
Effectiveness and safety of pulse oximetry in remote patient monitoring of patients with COVID-19: a systematic review, Alboksmaty *et al.*, 2022 [26^▪^].	2908 confirmed or presumptive COVID-19 patients.	Systematic review on effectiveness of SpO_2_% measurement combined with home monitoring systems.	Thirteen observational cohort studies were included, most during the first wave. Meta-analysis was not possible because of heterogeneity. Five studies used a mobile app or an online portal. Home monitoring was safe and showed potential to identify patients in need of advanced care. There was no hard evidence for the advantage of SpO_2_ in addition to standard monitoring programs.
The impact of posthospital remote monitoring of COVID-19 patients using pulse oximetry: A national observational study using hospital activity data, Georghiou *et al.*, 2022, United Kingdom [20^▪▪^].	139 619 suspected or confirmed COVID-19 patients.	Retrospective analysis using multivariate models. The goal was to assess the effectiveness of COVID Virtual Wards (CVW) in the United Kingdom on admissions duration and readmissions in the 28 days after discharge. Hospitals with CVW were compared with hospitals without CVW.All included patients were discharged between 17 August 2020 and 28 February 2021.	They found a longer hospital stay for patients discharged where CVW was available (incidence rate ratio 1.05, 95% CI 1.01–1.09). No relationship was found between CVW and readmission. Interestingly, not every patient discharged from the hospitals with CVW received home monitoring. In addition, home monitoring patients were sometimes still registered as admitted, thereby underestimating effectiveness.

## HOSPITAL CARE AT HOME: VIRTUAL WARD

COVID-19 can result in relative long hospitalization, especially because of the need for oxygen support [11]. Therefore, hospitals initiated early discharge of recovering COVID-19 patients to virtual wards to shorten physical hospitalization, especially from the second wave. Patients received oxygen and medications including dexamethasone and oral antibiotics at home. Earlier, we published our retrospective study about early discharge of 320 recovering COVID-19 patients (mean age 56 years) with home monitoring using a maximum of 3 l/min oxygen support starting from the first wave in the Netherlands. We calculated a 6 days reduction in hospitalization per patient on oxygen [12]. Another study showed even longer calculated reduction in hospitalization of 11 days per patients, using a similar protocol [13]. Both calculations were based on days of oxygen usage at home and, therefore, slower tapering at home probably resulted in an overestimation compared with in-hospital patients. An unblinded randomized controlled trial compared home monitoring with oxygen support with regular hospital care in 62 COVID-19 patients. During the 30-day follow-up, home monitoring reduced hospitalization with 1.7 days per patient. However, this was not statistically significant probably because of group size. Interestingly, physicians also discharged control group patients with oxygen, thereby reducing the chance to find significant differences between the intervention and control group. Moreover, control group patients requested early discharge resulting in faster tapering of 2 l/min in 1 day (see Table 2) [14]. In the United Kingdom, home monitoring of 43 COVID-19 patients with the majority receiving oxygen therapy at home (72%), resulted in shorter hospital stay compared with other admitted COVID-19 patients in the same period (10.3 vs. 11.9 days). However, the baseline characteristics of the patients not participating in home monitoring were unknown and could potentially clarify the differences (see Table 2) [15^▪^]. In contrast to the possible reduction in admission duration, home monitoring results in increased dependency on the manager of the home monitoring system. For example, patients were 4 days longer under hospital responsibility in the randomized controlled trial, because of slower tapering of oxygen and 24 h consideration time before inclusion [14]. To the best of our knowledge, all studies about home monitoring with oxygen deemed the system safe. Three to 12 percent presented to the emergency department, 3–9% readmitted and no fatalities occurred. Studies report high satisfaction rates for monitoring in their own home environment [12–14,15^▪^,^16^]. One study compared home monitoring combined with and without oxygen (up to 4 l/min) and found comparable readmission rates and mortality, which supports the safety of oxygen at home [17]. In conclusion, early discharge with home monitoring, oxygen and medication is safe. This may reduce hospitalization of COVID-19 patients. Although RCTs failed to show a significant reduction in hospitalization, which may have been related to trial design and small study populations.

## MONITORING RECOVERY

Recovery of COVID-19 patients was monitored following discharge without oxygen. Early detection of deterioration made intervention possible, to prevent readmission. In the United States, monitoring 225 relative young patients (age 41–69) without evident comorbidities after discharge resulted in a significant reduction of emergency department presentation [18]. Another study showed decrease in hospitalization for patients receiving home monitoring after discharge [19]. This is in contrast with a large retrospective analysis in the United Kingdom including 140 000 suspected or confirmed COVID-19 patients across the country. They analysed hospitalization duration and readmissions within 28 days after discharge and compared hospitals with and without home monitoring systems. Home monitoring resulted in a remarkable increase in hospitalization and no differences in readmissions. Nonetheless, not all patients discharged from the hospitals with home monitoring systems received home monitoring. In addition, home monitoring patients were sometimes still registered as admitted whilst in the monitoring program, making interpretation difficult [20^▪▪^]. The possible influence of home monitoring on hospitalization duration will be lower in patients without oxygen therapy. As COVID-19 admission may have major impact on patients, home monitoring after discharge from the hospital may offer support. This is highlighted by high satisfaction rates of home monitoring systems [4]. A study in the Netherlands investigated COVID-19 recovery for 6 months with home monitoring including home spirometry. As the study aimed to get insight in normal recovery, no interventions were performed, nor was a control group included. Forced vital capacity showed an ongoing linear recovery to 19% after 6 months and health status improved [21]. Monitoring systems for tele-rehabilitation by physiotherapists at home resulted in better physical recovery [22]. Tele-rehabilitation for pulmonary diseases is discussed in another chapter of this journal. To conclude, monitoring recovering COVID-19 patients without in-hospital treatments has low effectiveness but it can offer important support.

## HOME MONITORING REQUIREMENTS

Home monitoring for COVID-19 patients started throughout the world with often similar methods. Here, we will discuss requirements for home monitoring of COVID-19 patients. Regular assessment of the clinical condition is essential. Most systems combined vital signs and symptoms. Nurses or medical students performed daily triage and asked for alarming symptoms including dyspnoea, coughing or feeling more ill. Medical doctors supervised the consultations [9,23,24]. Sometimes, consultations were performed directly by the physician [5,25]. Monitoring centres made this process efficient and were accessible up to 24 h a day, 7 days a week. In some studies, every nurse could monitor 25–50 patients. In most cases, the hospital was responsible for the monitoring and sometimes the general practitioner (see Table 2) [26^▪^]. Communication methods included phone and video calls. Video enabled counting respiratory rate and general physical assessment [9]. Objective vital signs were crucial in addition to symptoms. Oxygen saturation at rest was the cardinal vital sign. One study used significant oxygen saturation drops (more than 4%) after 30 m walking to detect deterioration even earlier [24]. A systematic review showed that exertional desaturation needs further investigation [27]. The crucial role of oxygen saturation is explained by the pathophysiology of COVID-19. COVID-19 can cause a severe pneumonia with pulmonary oedema, endothelial wall thickening and dysfunctional alveolar–capillary oxygen transmission all causing hypoxemia. Therefore, about 75% of admitted patients eventually require supplemental oxygen [1]. One proposed characteristic feature of COVID-19 is the bad correlation between the severity of hypoxemia and dyspnoea, also known as silent hypoxemia. Patients do not feel dyspnoeic, although being severely hypoxemic [28,29]. In the absence of alarming symptoms, patients deteriorate and present in a late disease phase, sometimes requiring emergency intubation [30]. Pulse oximetry can detect hypoxemia and could, therefore, early deterioration. This was shown in a study including 77 COVID patients presenting to the emergency department with mild disease defined by oxygen saturation above 92%. They instructed patients to call if they desaturated or needed medical attention. Sixteen were re-admitted, including eight with asymptomatic low oxygen saturation accounting for the silent hypoxia [31]. However, the value of oxygen saturation in home monitoring is questioned by a recent systematic review published in the *Lancet*. Oxygen saturation measurement was well tolerated and could detect early deterioration, but there was lacking evidence for the benefit of oxygen saturation compared with regular consultations. Interestingly, there was a variation in the alarming threshold for oxygen saturation varying from 92 to 95% [26^▪^]. Other frequently measured parameters included respiratory rate, heart rate and temperature. Some studies measured blood pressure [4]. The additional value of every parameter other than oxygen saturation for monitoring COVID-19 has not been studied. Many different sensors are available for the measurement of combined vital signs at home. Skin patches can measure temperature, respiratory rate, pulse, blood pressure and oxygen saturation continuously [32]. Smart watches can register heart rate and activity level [33]. An in-ear device can measure SpO_2_, respiratory rate, heart rate and temperature [34]. The field of sensors is under fast development. Hospitals also use these sensors for monitoring during admissions, for example, in isolation rooms to reduce patient contact for nurses.

Home monitoring programs used a variety of tablet or mobile phone applications to register data. The apps could instruct patients to perform measurements or fill out symptom questionnaires. Some apps also informed patients about the disease or gave practical tips for recovery [35]. Applications used thresholds to send alarms to the healthcare professionals, for example, if there was an increase in dyspnoea or oxygen saturation below 92%. The professional could thereafter perform a call and escalate care if necessary. The use of an app in combination with phone calls reduced the workload in a study in the UK [36]. Patient satisfaction of an app was comparable to video consultation, about 80% [37]. In one study, special COVID watches sent automated messages twice a day asking if patients felt worse or dyspnoeic. If they answered both with yes, or if they texted ‘worse’, a nurse performed a call and could escalate care. Compared with a control group, this resulted in higher consultation rates, more emergency department presentation and lower mortality [38].

## DISCUSSION

**FIGURE 2:**
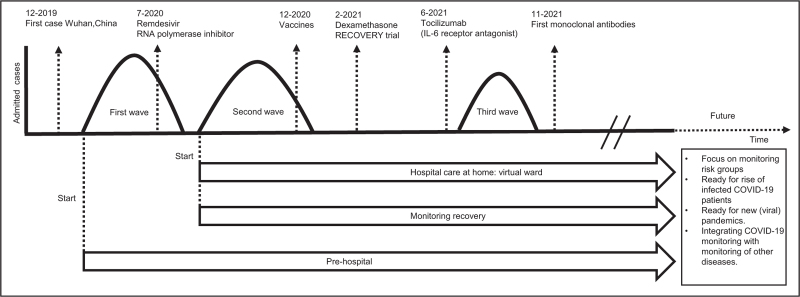
Schematic simplified overview of COVID-19 treatment and home monitoring systems over time. COVID-19 disease changed over time because of development of vaccines and medication (dates are according to European Medicines Agency or United States Food and Drug Administration authorization). Home monitoring developed over time as well. During the first wave monitoring of prehospital patients started, meaning no previous admission except for an emergency department presentation. From the second wave, early discharge of patients with hospital care at home (oxygen and medication) and monitoring recovery without in-hospital treatment started. In the future, monitoring systems should focus on risk groups. They should be ready for new (viral) pandemics and integrate with home monitoring for other diseases. IL, interleukin.

During the COVID-19 pandemic, many different home monitoring systems were investigated. Most studies selected young patients with few comorbidities, due to requirement of digital skills and risk avoidance. As a consequence, after invitation for home monitoring, activation was highest among young, female and white patients thereby also selecting low-risk population [39]. This probably led to less cost-effectiveness because of intensive costly monitoring with less interventional needs. In the future, selection of high-risk COVID-19 patients, for example, kidney transplant and other immunocompromised patients, could increase effectiveness [40,41]. Scoring systems could select high-risk patients, for example, based on dyspnoea, sex, temperature and hypertension [42]. Home monitoring systems must be adapted to this population. This also meets the current healthcare needs. Algorithms could increase efficiency by predicting the need for clinical assessment and escalation of care, for example, by using age, degree of breathlessness, fatigue and oxygen saturation at rest or predict desaturation by serial oxygen saturation and heart rate monitoring [42,43]. However, these algorithm first need to be tested among different patient groups. During the first wave in 2020, the COVID-19-related healthcare demand was higher than in the current postvaccine era with ever improving treatments (see Fig. 2). The influence of vaccination and treatment on COVID-19 makes studies performed at different time points hard to compare [6]. In earlier studies, the advantage of a home monitoring would probably be higher because of higher disease burden. As COVID-19 caseload fluctuates, monitoring systems must be adaptable to the needs of the healthcare system. Monitoring systems should be ready for new pandemics and should integrate with home monitoring systems for other diseases to increase efficiency.

## CONCLUSION

Home monitoring programs have been developed for different disease phases of COVID-19 and showed great safety and patient satisfaction. The best strategy depends on the patient population and proposed phase of the COVID-19 disease. In the prehospital phase, home monitoring reduces hospitalization by early detection of deterioration and intervention. Discharge with oxygen support and oral medication probably results in shorter hospitalization but longer care under hospital responsibility. At last, home monitoring can offer support in the recovery process. The status of the pandemic influenced the need for home monitoring.

## Acknowledgements

*None.*

### Financial support and sponsorship

*None.*

### Conflicts of interest

*A.V. received consulting fees from Boehringer Ingelheim.*

## REFERENCES AND RECOMMENDED READING

Papers of particular interest, published within the annual period of review, have been highlighted as:

▪ of special interest
▪▪ of outstanding interest
